# ACOT1 deficiency attenuates high-fat diet–induced fat mass gain by increasing energy expenditure

**DOI:** 10.1172/jci.insight.160987

**Published:** 2023-09-22

**Authors:** Timothy D. Heden, Mallory P. Franklin, Christina Dailey, Mara T. Mashek, Chen Chen, Douglas G. Mashek

**Affiliations:** 1 Pennington Biomedical Research Center, Baton Rouge, Louisiana, USA.; 2 Department of Biochemistry, Molecular Biology and Biophysics and; 3 Department of Medicine, Division of Diabetes, Endocrinology and Metabolism, Medical School, University of Minnesota, Minneapolis, Minnesota, USA.

**Keywords:** Metabolism, Fatty acid oxidation, Obesity, Uncoupling proteins

## Abstract

Acyl-CoA thioesterase 1 (ACOT1) catalyzes the hydrolysis of long-chain acyl-CoAs to free fatty acids and CoA and is typically upregulated in obesity. Whether targeting ACOT1 in the setting of high-fat diet–induced (HFD-induced) obesity would be metabolically beneficial is not known. Here we report that male and female ACOT1KO mice are partially protected from HFD-induced obesity, an effect associated with increased energy expenditure without alterations in physical activity or food intake. In males, ACOT1 deficiency increased mitochondrial uncoupling protein-2 (UCP2) protein abundance while reducing 4-hydroxynonenal, a marker of oxidative stress, in white adipose tissue and liver of HFD-fed mice. Moreover, concurrent knockdown (KD) of UCP2 with ACOT1 in hepatocytes prevented increases in oxygen consumption observed with ACOT1 KD during high lipid loading, suggesting that UCP2-induced uncoupling may increase energy expenditure to attenuate weight gain. Together, these data indicate that targeting ACOT1 may be effective for obesity prevention during caloric excess by increasing energy expenditure.

## Graphical abstract

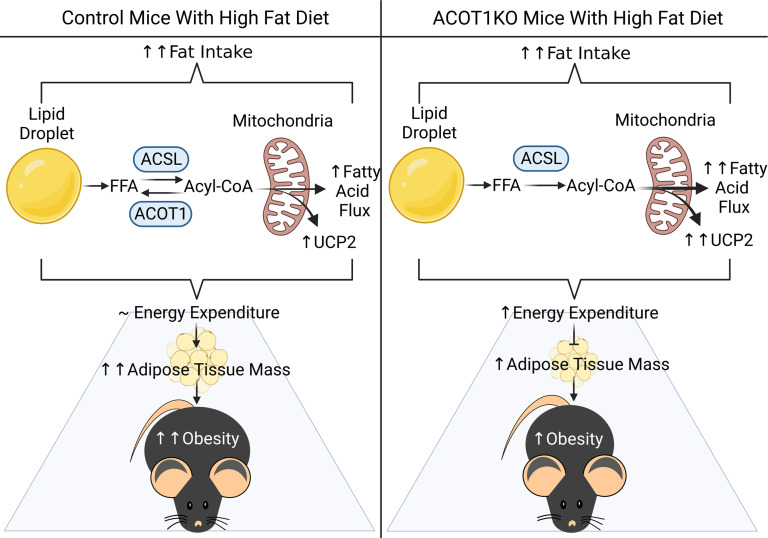

## Introduction

Obesity impacts a large proportion of society and is associated with several comorbidities, including heart disease, type 2 diabetes, and nonalcoholic fatty liver disease (1). A major contributor to the development of obesity and comorbidities is excess free fatty acids (FAs) (2), derived from the diet or synthesized de novo, which partake in a wide array of processes in cell metabolism and signaling. Before being metabolized, free FAs must be activated by long-chain acyl-CoA synthetases to acyl-CoAs (3), which can subsequently proceed into diverse metabolic pathways, including glycerolipid and sphingolipid synthesis, esterification to retinol and cholesterol, or conversion to acylcarnitine for mitochondrial β-oxidation (4). An alternative metabolic fate for acyl-CoAs is the reversion back to free FAs and CoA via acyl-CoA thioesterase (ACOT) enzymes (3, 5). It is presumed that this reaction helps maintain proper ratios of free and activated FAs as well as CoA levels (3, 5), although the physiological importance of ACOT enzymes in metabolism has not been extensively characterized. The ACOT enzymes fall within 2 structurally distinct families in mammals — type I (ACOT 1–6) and II (ACOT 7–13) proteins (6), with each having various degrees of expression patterns in tissues, localizing with different organelles, and hydrolyzing a broad range of acyl-CoA species (3, 5). ACOT1 is a cytosolic and nuclear thioesterase (3, 5, 7) that shows increased expression in various tissues, such as the liver, in response to fasting or a high-fat diet (HFD) (7–9). ACOT1 expression is downregulated in differentiating brown adipocytes (10) but upregulated in brown adipose tissue (BAT) in response to cold stress (8). Prior work from our lab showed that acute knockdown (KD) of ACOT1 in the liver enhances FA oxidation and reduces hepatic triglyceride levels (7). A recent study using an ATAC-Seq analysis found that hepatic ACOT1 is elevated and deregulated in individuals with type 2 diabetes (11). Although these hepatic specific effects have been described, the impact of prolonged, global loss of ACOT1 on whole-body metabolism has not been characterized. To address this knowledge gap, this study characterizes the effects of global ACOT1KO on lipid homeostasis in mice fed a low-fat diet (LFD) or HFD. We find that ACOT1KO partially protects male and female mice from HFD-induced fat weight gain, effects that were associated with increased mitochondrial uncoupling protein-2 (UCP2) protein abundance and energy expenditure.

## Results

### ACOT1KO attenuates HFD-induced weight gain.

**Figure 1:**
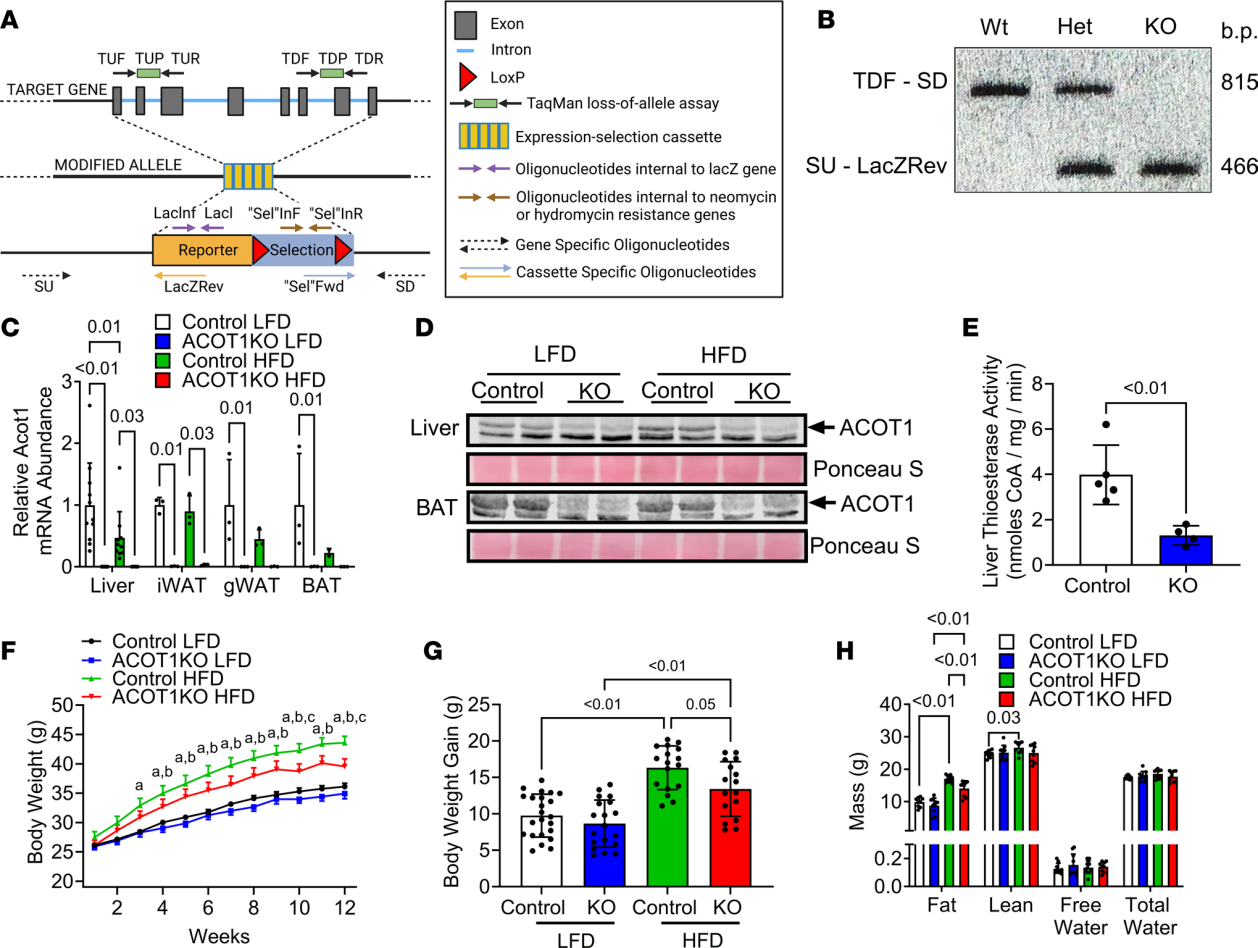
ACOT1KO modestly attenuates HFD-induced fat weight gain in male mice. (**A**) VelociGene KOMP null allele design. (**B**) Southern blot image showing WT, heterozygous (Het), and homozygous (KO) bands for genotyping. (**C**) ACOT1 mRNA abundance in liver, iWAT, gWAT, and BAT in 20-week-old control male mice fed the LFD for 12 weeks, ACOT1KO mice fed the LFD for 12 weeks, control mice fed the HFD for 12 weeks, and ACOT1KO mice fed the HFD for 12 weeks (*n* = 2–11 per group). (**D**) Western blot images of ACOT1 in liver and BAT tissues (*n* = 2 per group). (**E**) Liver thioesterase activity (*n* = 4–5 per group). (**F**) BW time course (*n* = 16–24 per group). (**G**) BW gain during each diet (*n* = 16–23 per group). (**H**) Echo MRI body composition data (*n* = 9–10 per group). All data are presented as mean ± SEM. An ANOVA (**G** used 1 way; **C**, **D**, **F**, and **H** used a 2 way) or 2-tailed *t* test (**E**) was used for statistical tests. ^a^*P* ≤ 0.01 control LFD versus control HFD, ^b^*P* = 0.02 ACOT1KO LFD versus ACOT1KO HFD, and ^c^*P* ≤ 0.01 control HFD versus ACOT1KO HFD.

Mice with whole body ACOT1KO were purchased from the NIH Knockout Mouse Project (VG12838), which uses VelociGene’s KOMP Allele Genotyping Strategies (Figure 1A). ACOT1KO via PCR was confirmed by the detection of the cassette SU-LacZRev (Figure 1B). Additionally, ACOT1KO mice had undetectable levels of *Acot1* mRNA levels in liver, inguinal (s.c.) white adipose tissue (iWAT), gonadal WAT (gWAT), and BAT (Figure 1C), in addition to undetectable ACOT1 protein levels in liver and BAT (Figure 1D and Supplemental Figure 1A; supplemental material available online with this article; https://doi.org/10.1172/jci.insight.160987DS1) and reduced liver thioesterase activity (Figure 1E). The HFD reduced the mRNA abundance of *Acot1* approximately 50% in liver, but it did not significantly impact *Acot1* mRNA abundance in other tissues (Figure 1C), while ACOT1 protein abundance was higher in the liver of HFD-fed mice (Figure 1D and Supplemental Figure 1A). Over the course of the 12-week HFD, male ACOT1KO mice gained less BW (Figure 1, F and G) compared with male control mice given the HFD, and this attenuation of weight gain was due to less fat mass accretion without alterations in lean mass, free water, or total water (Figure 1H). Female mice displayed a similar phenotype and had attenuated HFD-induced weight gain and fat mass accretion (Supplemental Figure 1, B–D). Together, these results indicate that ACOT1 deficiency attenuates the increase in adiposity in response to HFD feeding.

### Increased energy expenditure in ACOT1KO mice fed the HFD.

**Figure 2:**
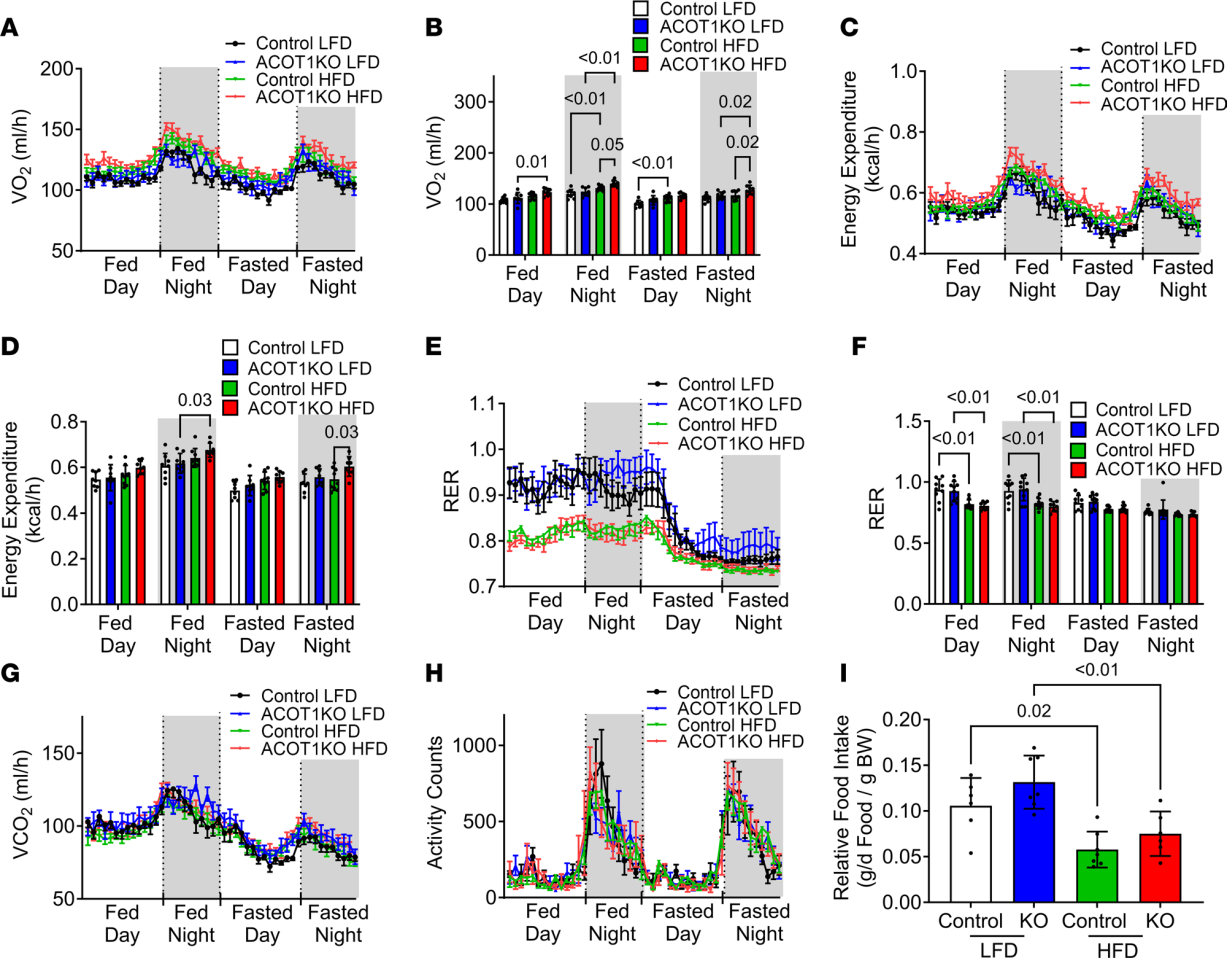
ACOT1KO modestly increases energy expenditure. (**A**) Oxygen consumption (VO_2_) time course during the light and dark cycle in 20-week-old control or ACOT1KO mice given the LFD or HFD for 12 weeks (*n* = 8–9 per group). (**B**) Average VO_2_ during the light and dark cycle (*n* = 8–9 per group). (**C**) Energy expenditure time course during the light and dark cycle (*n* = 8–9 per group). (**D**) Average energy expenditure during the light and dark cycle (*n* = 8–9 per group). (**E**) RER time course during the light and dark cycle (*n* = 8–9 per group). (**F**) Average RER during the light and dark cycle (*n* = 8–9 per group). (**G**) Carbon dioxide production (VCO_2_) time course during the light and dark cycle (*n* = 8–9 per group). (**H**) Activity counts time course during the light and dark cycle (*n* = 8–9 per group). (**I**) Relative food intake (*n* = 6–7 per group). The indirect calorimetry time course figures are 48 hours in length, and during the first 24 hours, the mice were fed and had free access to food, while during the last 24 hours, the mice were fasted without food but had free access to water. The data are presented as mean ± SEM. ANCOVA was used for statistical tests.

The primary mechanisms most likely underlying the reduction in fat weight gain in ACOT1KO mice fed the HFD are increased energy expenditure, reduced food intake, or a combination of both. To determine if ACOT1KO modulated oxygen consumption or food intake, mice were housed in metabolic cages, and indirect calorimetry was used to measure oxygen consumption, carbon dioxide production, and substrate metabolism with simultaneous measurements of physical activity and food intake. Male ACOT1KO mice fed the HFD displayed higher oxygen consumption (Figure 2, A and B) and energy expenditure (Figure 2, C and D) rates, particularly during fasting and the dark cycle. Moreover, oxygen consumption tended to be higher in ACOT1KO mice fed the HFD compared with control mice fed the HFD during the feeding period and during the dark cycle (*P* = 0.08). The increase in oxygen consumption in ACOT1KO mice fed the HFD was not associated with alterations in substrate metabolism, i.e., respiratory exchange ratio (RER) (Figure 2, E and F), carbon dioxide production (Figure 2G), physical activity counts (Figure 2H), or food intake (Figure 2I), indicating that ACOT1KO modestly promotes an increase in energy expenditure during HFD feeding, which likely contributes to attenuated fat mass gain.

### ACOT1 deficiency, glucose tolerance, and insulin sensitivity.

Prevention of fat weight gain with HFD feeding is often associated with enhanced glucose tolerance and insulin sensitivity. Although ACOT1KO modestly reduced fat weight gain in male mice fed an HFD, there were no significant differences in glucose tolerance (Supplemental Figure 2, A and B). An insulin tolerance test was used to assess insulin sensitivity, and the HFD reduced insulin sensitivity as indicated by greater blood glucose concentrations 15 minutes after insulin injection in HFD-fed male mice compared with LFD-fed mice (Supplemental Figure 2C). Moreover, 90 minutes after insulin injection, the ACOT1KO mice had modestly lower blood glucose levels compared with the control animals, suggesting higher insulin sensitivity (Supplemental Figure 2C), although the insulin incremental AUC was not significantly different between groups (Supplemental Figure 2D). Moreover, fasting insulin (Supplemental Figure 2E) or insulin resistance measured via homeostatic model assessment of insulin resistance (HOMA-IR) (Supplemental Figure 2F) was not significantly different between groups. In female mice, no differences in glucose tolerance between groups were observed (Supplemental Figure 3, A and B). Although glucose levels during the end of the insulin tolerance test were higher in control female mice fed the HFD compared with control female mice fed the LFD, no other differences in parameters for insulin sensitivity were observed (Supplemental Figure 3, C–F).

### ACOT1KO increases mitochondrial UCP2 in BAT from LFD- but not HFD-fed mice.

**Figure 3:**
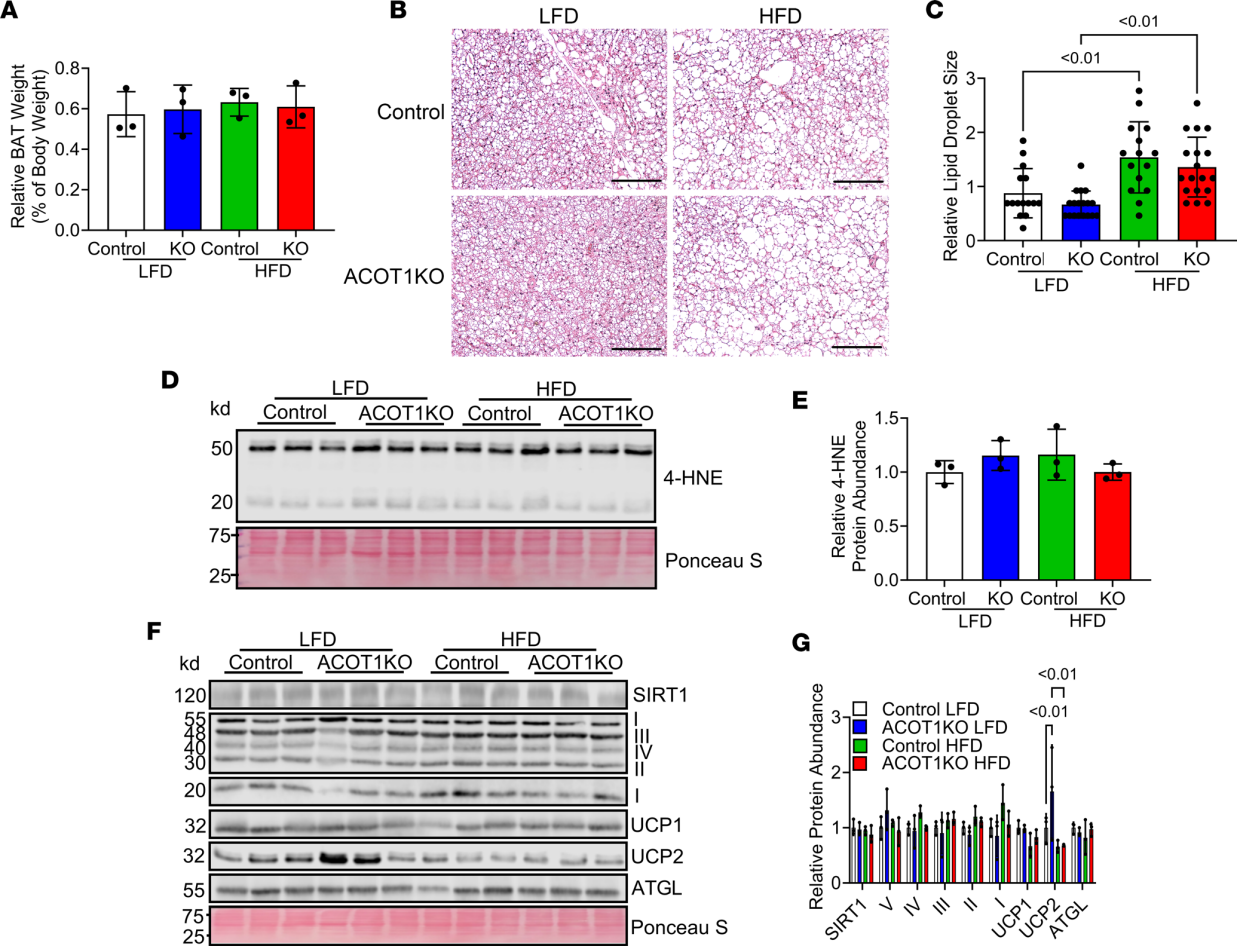
ACOT1KO increases mitochondrial UCP2 abundance in BAT of LFD-fed male mice. (**A**) Relative BAT weight in 20-week-old control or ACOT1KO mice given the LFD or HFD for 12 weeks (*n* = 3 per group). (**B**) H&E staining of BAT tissue sections (representative images shown, images from *n* = 3 mice per group were taken; scale bar: 100 μm). (**C**) Average adipocyte size in H&E images (*n* = 15–18 per group). (**D**) Western blot image of 4-HNE, a marker of oxidative stress (*n* = 3 per group). (**E**) Densitometry quantification of images in **D** (*n* = 3 per group). (**F**) Western blot image of SIRT1, Complex I-V of the electron transport chain, UCP1 and 2, and ATGL (*n* = 3 per group). Ponceau S was used to assess protein loading for every blot, but only a representative image is shown. (**G**) Densitometry quantification of images in **F** (*n* = 3 per group). All data are presented as mean ± SEM. ANOVA was used for statistical tests.

BAT is responsible for generating heat to maintain body temperature, and in response to cold exposure, BAT ACOT1 abundance increases (8). Targeting BAT to increase oxygen consumption via mitochondrial UCP1 has been studied as a strategy to prevent or treat obesity; thus, we next determined if ACOT1KO modified BAT metabolism in a manner that would increase oxygen consumption to attenuate fat weight gain during the HFD. ACOT1KO did not significantly modify BAT weight (Figure 3A), tissue morphology, lipid droplet size (Figure 3, B and C), or the protein abundance of 4-hydroxynonenal (4-HNE), a marker of lipid peroxidation and ROS, or UCP1 (Figure 3, F and G) in LFD- or HFD-fed animals, suggesting that some other mechanism may contribute to increased oxygen consumption in ACOT1KO mice fed the HFD. Thus, it was next determined if ACOT1KO modified the protein abundance of key enzymes involved in regulating mitochondrial oxygen consumption or ROS. Sirtuin 1 (SIRT1) protein abundance, which is an NAD^+^-dependent protein deacetylase involved in regulating mitochondrial biogenesis, was not modified (Figure 3, F and G). Complex I-V of the electron transport chain, or adipose triglyceride lipase (ATGL), which are markers of mitochondrial content and lipolysis, respectively, were not modified by ACOT1KO or HFD (Figure 3, F and G). ACOT1KO might impact UCP1-independent pathways of thermogenesis to increase energy expenditure. To test this, the protein abundance of sarcolipin (SLN), a protein that uncouples the sarcoplasmic/endoplasmic reticulum Ca^2+^-ATPase pump to increase nonshivering thermogenesis in skeletal muscle (12), was measured, but no significant differences between groups were observed (Supplemental Figure 4A). Moreover, the protein abundance of glycine amidinotransferase (GATM), the rate-limiting enzyme in creatine synthesis that has a role in diet-induced thermogenesis in adipose tissue (13), was measured in BAT (Supplemental Figure 4B), gWAT (Supplemental Figure 4C), and iWAT (Supplemental Figure 4D) tissues. In BAT, an HFD increased GATM protein levels in control mice but not ACOT1KO mice. In gWAT and iWAT, GATM protein abundance was lower in ACOT1KO mice fed the LFD and tended to be lower in animals given the HFD. Together, given that ACOT1KO did not increase SLN in skeletal muscle or GATM in various adipose tissue depots in mice fed the HFD, these data suggest that UCP1-independent mechanisms of thermogenesis likely do not contribute to the increase in energy expenditure observed. Curiously, ACOT1KO mice fed the LFD had higher UCP2 protein abundance compared with control animals fed the LFD, as well as ACOT1KO mice fed the HFD (Figure 3, F and G), suggesting that ACOT1KO may impact UCP2. Collectively, these data suggest that the increased oxygen consumption observed with HFD is not due to alterations in the classical BAT UCP1-mediated thermogenic pathway or UCP1-independent thermogenic pathways in skeletal muscle or WAT.

### ACOT1KO reduces WAT mass, lipid droplet size, and oxidative stress.

**Figure 4:**
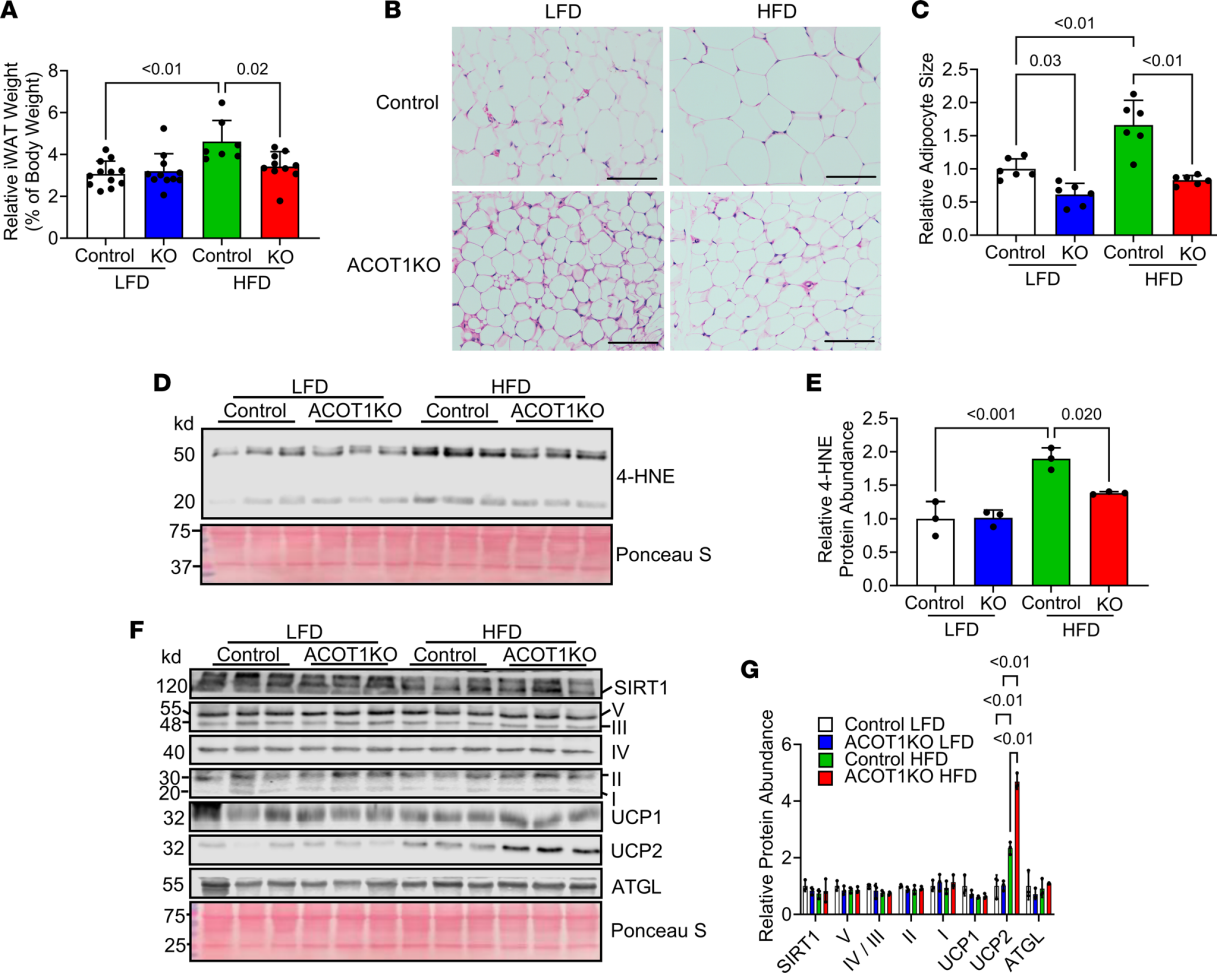
ACOT1KO reduces iWAT size and oxidative stress in HFD-fed male mice. (**A**) Relative iWAT weight in 20-week-old control or ACOT1KO mice given the LFD or HFD for 12 weeks (*n* = 7–12 per group). (**B**) H&E staining of iWAT tissue sections (representative images shown, images from *n* = 3 mice per group were taken; scale bar: 100 μm). (**C**) Average adipocyte size in H&E images (*n* = 6 per group). (**D**) Western blot image of 4-HNE, a marker of oxidative stress (*n* = 3 per group). (**E**) Densitometry quantification of images in **D** (*n* = 3 per group). (**F**) Western blot image of SIRT1, Complex I-V of the electron transport chain, UCP1 and 2, and ATGL (*n* = 3 per group). Ponceau S was used to assess protein loading for every blot, but only a representative image is shown. (**G**) Densitometry quantification of images in **F** (*n* = 3 per group). All data are presented as mean ± SEM. ANOVA was used for statistical tests.

Given that ACOT1 deficiency modestly reduced fat mass gain, we next explored if WAT was modified in a manner that would prevent fat weight gain during HFD feeding. ACOT1 deficiency reduced iWAT (Figure 4A) weight during the HFD, an effect that was associated with reduced lipid droplet size (Figure 4, B and C). Additionally, the iWAT protein abundance of 4-HNE, a marker of oxidative stress, was modestly lower in ACOT1KO mice fed an HFD compared with control mice fed an HFD (Figure 4, D and E), and this coincided with an increase in protein abundance of UCP2, without alterations in the protein abundance of UCP1 or markers of mitochondrial metabolism (SIRT1, Complex I-V of electron transport chain), or lipolysis (ATGL) (Figure 4, F and G). Some studies show that UCP2 overexpression increases mitochondrial uncoupling (14), suggesting that increased oxygen consumption during HFD feeding in ACOT1KO mice may be mediated via UCP2.

**Figure 5:**
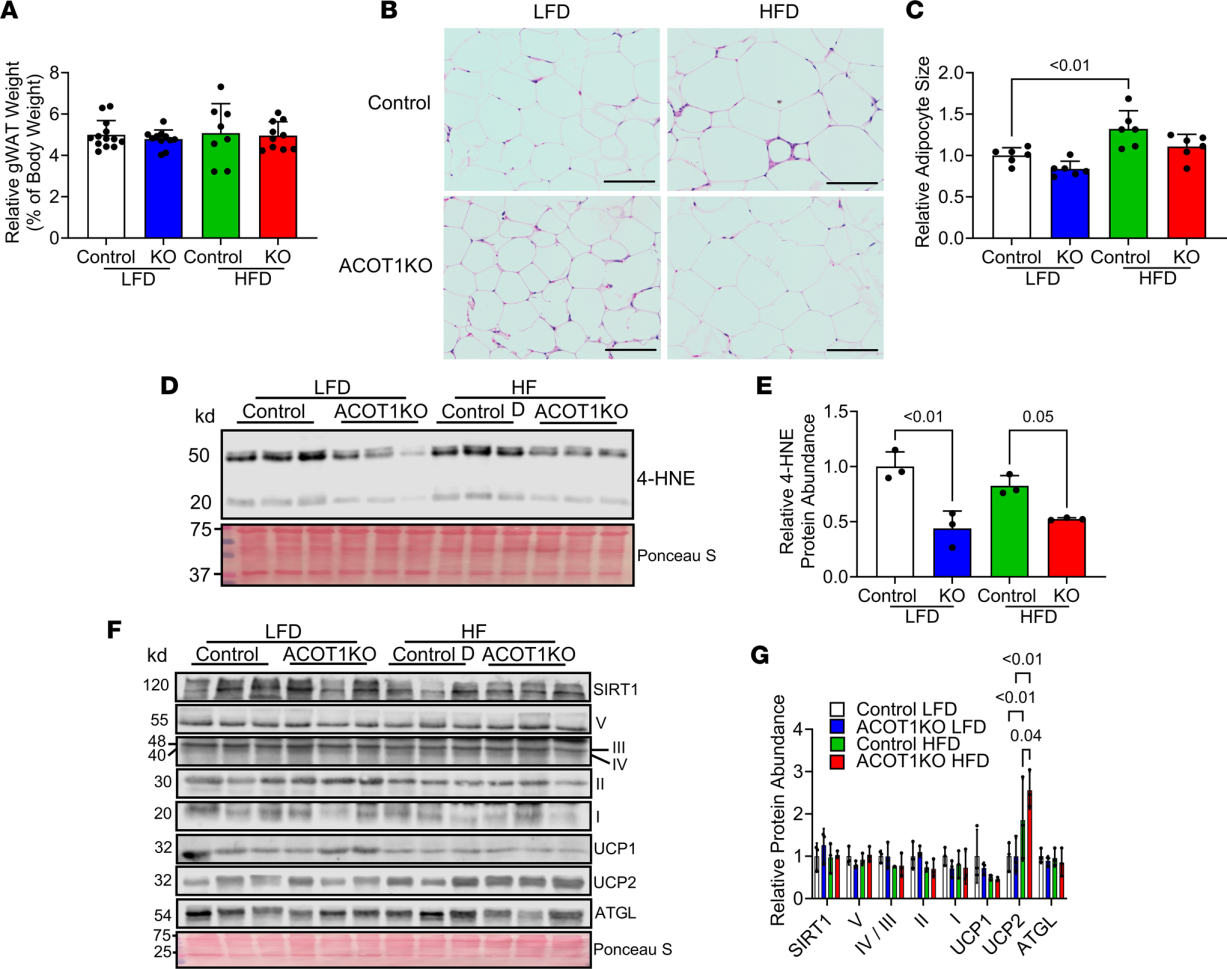
ACOT1KO reduces gWAT size and oxidative stress in HFD-fed male mice. (**A**) Relative gWAT weight in 20-week-old control or ACOT1KO mice given the LFD or HFD for 12 weeks (*n* = 8–13 per group). (**B**) H&E staining of gWAT tissue sections (representative images shown, images from *n* = 3 mice per group were taken; scale bar: 100 μm). (**C**) Average adipocyte size in H&E images (*n* = 6 per group). (**D**) Western blot image of 4-HNE, a marker of oxidative stress (*n* = 3 per group). (**E**) Densitometry quantification of images in **D** (*n* = 3 per group). (**F**) Western blot image of SIRT1, Complex I-V of the electron transport chain, UCP1 and 2, and ATGL (*n* = 3 per group). Ponceau S was used to assess protein loading for every blot, but only a representative image is shown. (**G**) Densitometry quantification of images in **F** (*n* = 3 per group). All data are presented as mean ± SEM. ANOVA was used for statistical tests.

Despite no differences in tissue weight (Figure 5A), the HFD increased gWAT adipocyte size in control mice, whereas adipocyte size was not significantly modified with the HFD in ACOT1KO mice (Figure 5, B and C). Consistent with what was observed in iWAT, 4-HNE protein abundance was reduced (Figure 5, D and E), while UCP2 protein abundance was increased, in gWAT tissue from ACOT1KO mice fed the HFD; in contrast, other markers of mitochondrial metabolism (SIRT1, Complex I-V of electron transport chain), or lipolysis (ATGL) were not altered (Figure 5, F and G).

**Figure 6:**
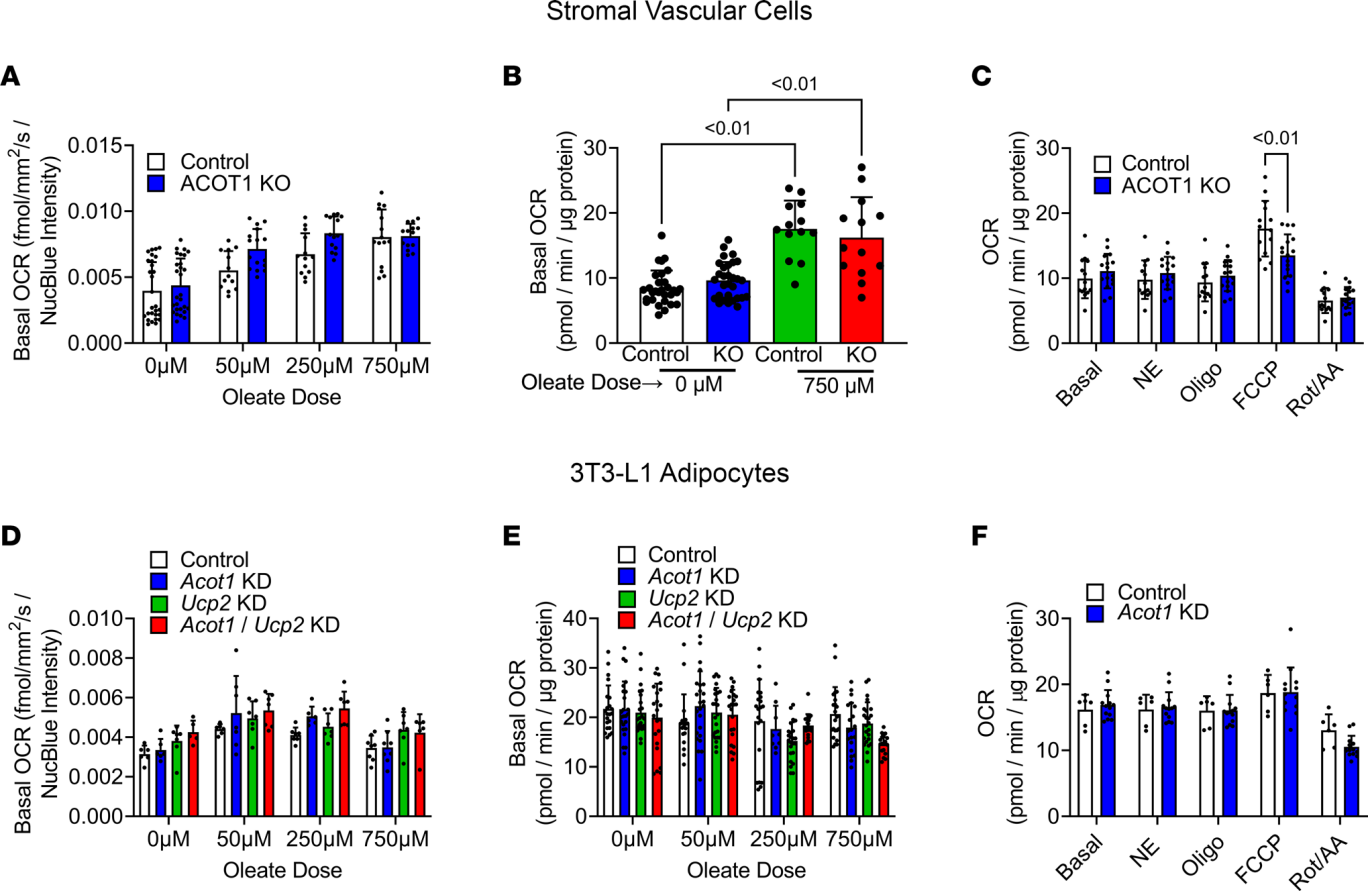
ACOT1KO and respiration in isolated adipocytes. (**A**) Basal OCR in stromal vascular cells were measured over approximately a 22- to 24-hour time period with a Resipher system (*n* = 13–28 per group). (**B**) Basal OCR in stromal vascular cells was measured over an approximately 20-minute period with a Seahorse analyzer (*n* = 13–30 per group). (**C**) OCR in stromal vascular cells was measured with a Seahorse under basal conditions and after sequential injections of norepinephrine (NE), oligomycin (oligo), FCCP, and rotenone/antimycin A (Rot/AA) (*n* = 14–16 per group). (**D**) Basal OCR in 3T3-L1 cells treated with a scrambled (control), sh*Acot1*, sh*Ucp2*, or both sh*Acot1*/sh*Ucp2* lentivirus to induce gene KD (*n* = 5–8 per group). (**E**) Basal OCR in 3T3-L1 cells was measured over an approximately 20-minute period with a Seahorse analyzer (*n* = 9–24 per group). (**F**) OCR in 3T3-L1 cells was measured with a Seahorse under basal conditions and after sequential injections of NE, oligo, FCCP, and Rot/AA (*n* = 6–13 per group). ANOVA was used for statistical tests.

To mechanistically explore if ACOT1 deficiency increased energy expenditure specifically in adipocytes to increase whole-body energy expenditure, stromal vascular cells isolated from control or ACOT1KO mice were differentiated into adipocytes, and oxygen consumption rates (OCR) were measured with a Resipher or Seahorse XFe96 analyzer under various oleate loads (Figure 6, A and B). Although greater amounts of exogenous lipid increased OCR, there were no significant differences between control and ACOT1KO cells (Figure 6, A and B). To determine if norepinephrine (NE) or maximal carbonyl cyanide-*p*-trifluoromethoxyphenyl hydrazone (FCCP) stimulation was impacted by ACOT1KO, a modified Seahorse mitochondrial stress test was performed in which OCR was measured after sequential additions of NE, oligomycin, FCCP, and rotenone with antimycin A (Figure 6C). ACOT1KO reduced maximal FCCP-stimulated OCR, while respiration rates by NE were not impacted. Similarly, ACOT1KO in stromal vascular cells reduced maximal FCCP-stimulated OCR during a modified Seahorse mitochondrial stress test with 8-bromo-cAMP instead of NE, as described above (Supplemental Figure 5A). The role of UCP2 in thermogenesis and mitochondrial uncoupling in adipocytes is controversial. Thus, we next explored how UCP2 KD and simultaneous UCP2 and ACOT1 KD impacted OCR under various oleate loads in 3T3-L1 cells (Figure 6, D and E). Despite a robust KD in UCP2 or both ACOT1 and UCP2 (Supplemental Figure 5B), neither UCP2 KD nor ACOT1/UCP2 double KD impacted OCR during different oleate loads measured with a Resipher (Figure 6D) or Seahorse (Figure 6E) system. Furthermore, NE stimulation in 3T3-L1 adipocytes with ACOT1 KD did not differentially impact OCR (Figure 6F). Together, these data suggest that the increase in whole-body energy expenditure in ACOT1KO mice consuming an HFD may not be due to alterations in WAT energy expenditure.

### ACOT1KO increases hepatic FA oxidation and mitochondrial uncoupling.

**Figure 7:**
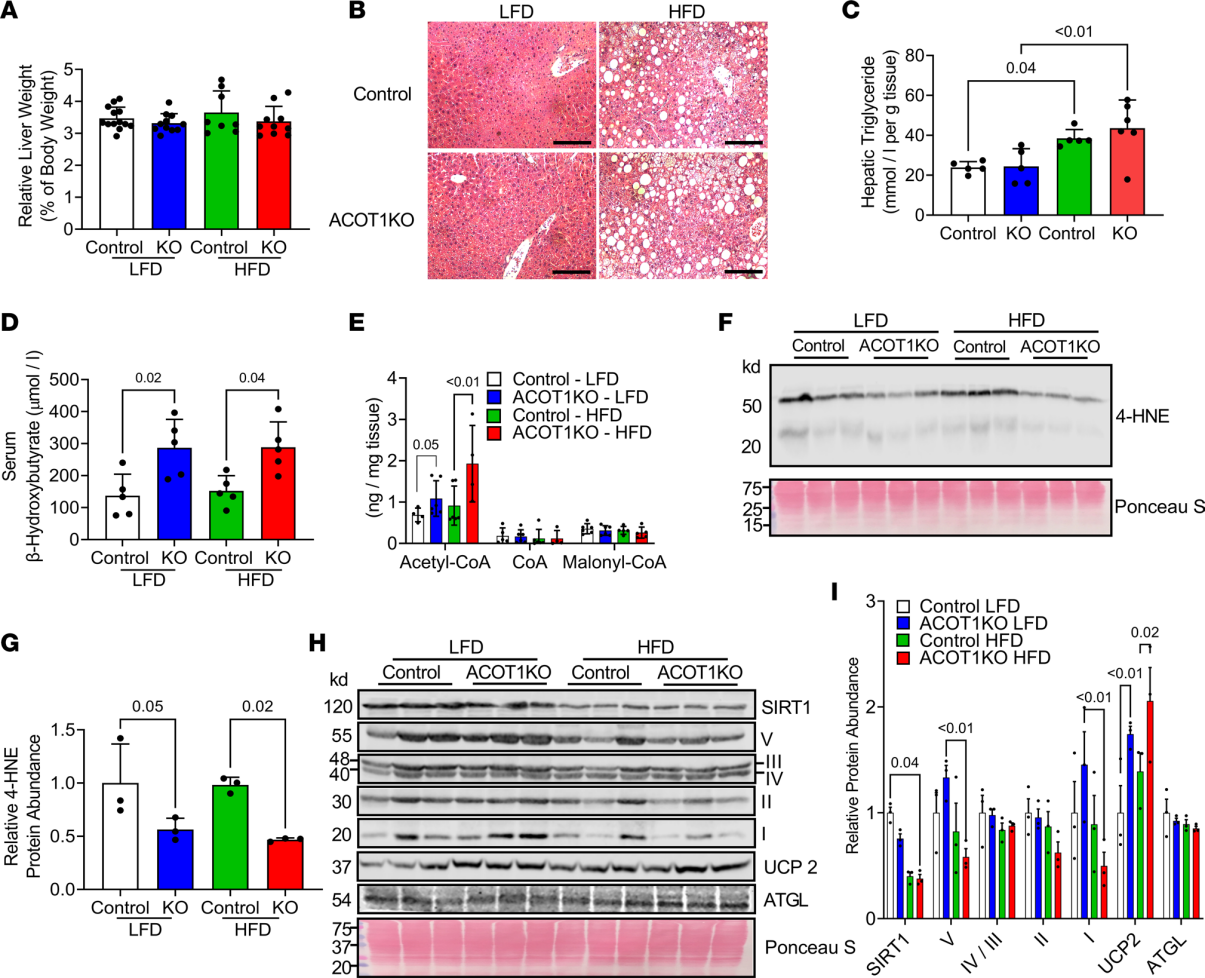
Reduced oxidative stress and increased FA oxidation and mitochondrial UCP2 in ACOT1KO livers. (**A**) Relative liver weights in 20-week-old control or ACOT1KO mice given the LFD or HFD for 12 weeks (*n* = 8–13 per group). (**B**) H&E staining of liver tissue sections (representative images shown, images from *n* = 3 mice per group were taken; scale bar: 100 μm). (**C**) Liver triglyceride levels (*n* = 5–6 per group). (**D**) Serum β-hydroxybutyrate levels (*n* = 5 per group). (**E**) Hepatic acyl-CoA levels (*n* = 4–6 per group). (**F**) Western blot image of 4-HNE, a marker of oxidative stress and protein carbonylation (*n* = 3 per group). (**G**) Densitometry quantification of upper and lower bands in F (*n* = 3 per group). (**H**) Western blot images of SIRT1, Complex I-V of the electron transport chain, UCP2, and ATGL (*n* = 3 per group). Ponceau S was used to assess protein loading for every blot, but only a representative image is shown. (**I**) Densitometry quantification of images in **H** (*n* = 3 per group). All data are presented as mean ± SEM. ANOVA was used for the statistical tests.

Obesity and excess BW gain increase lipid deposition in peripheral tissues, particularly the liver. Given that ACOT1KO modestly attenuated HFD-induced weight gain and reduced adipose tissue weight, it was next determined if hepatic triglyceride content was modified with ACOT1KO. There were no differences in relative liver weight (Figure 7A), while H&E staining (Figure 7B) in conjunction with measurements of liver triglyceride levels (Figure 7C) revealed that the HFD increased hepatic lipid stores but that ACOT1KO did not modify this effect. Primary routes of disposal for liver triglyceride are secretion out of the liver and into circulation as part of very low-density lipoprotein particles or through oxidation of liberated FAs via β-oxidation (15). ACOT1KO mice did not show alterations in serum triglyceride levels (Supplemental Figure 6A) but ACOT1KO mice, independent of diet, had elevated serum β-hydroxybutyrate levels (Figure 7D) and liver acetyl-CoA levels (Figure 7E), suggesting that ACOT1 deficiency increased flux of hepatic FAs toward oxidation, despite not altering triglyceride levels. Fatty acid oxidation within mitochondria generates ROS, and despite an increase in FA oxidation, ACOT1KO mice fed the HFD displayed lower hepatic ROS as indicated by a significant reduction in 4-HNE protein abundance (Figure 7, F and G), an effect that was independent of diet. The major cellular antioxidant in the liver is glutathione (GSH), but we did not find any significant difference in the abundance of reduced GSH or oxidized GSH (Supplemental Figure 6B), suggesting that some other factor is contributing to the reduction in ROS with ACOT1KO during HFD feeding. Fatty acid oxidation and oxidative stress in the liver can be modulated through various proteins involved in coordinating energy homeostasis. Thus, it was next determined if ACOT1KO modified the protein abundance of key enzymes involved in regulating mitochondrial energy metabolism or oxidative stress in the liver. HFD reduced SIRT1 protein abundance to a similar extent in both control and ACOT1KO mice (Figure 7, H and I). Despite an increase in FA oxidation, when challenged with the HFD, ACOT1KO animals had lower mitochondrial Complex I and Complex V protein abundance compared with ACOT1KO animals fed the LFD, suggesting a reduction in only select components of the mitochondrial electron transport chain (Figure 7, H and I). However, mitochondrial DNA (mtDNA) content, as measured by the ratio of ND2 mtDNA/HK2 nuclear DNA or 16s rRNA mtDNA/HK2 nuclear DNA, was not significantly modified, indicating that overall mitochondrial content did not dramatically change because of the diet or genotype (Supplemental Figure 6C). Consistent with what was observed in WAT from ACOT1KO mice fed the HFD, UCP2 protein abundance was also greater in the liver of ACOT1KO mice fed the HFD while ATGL content was not modified (Figure 7, H and I). These changes in protein data were consistent with changes in mRNA as *Ucp2* mRNA abundance was highest in the ACOT1KO mice fed the HFD (Supplemental Figure 6D), suggesting that UCP2 may mediate the increase in oxygen consumption observed in ACOT1KO mice fed the HFD.

### ACOT1 KD increases oxygen consumption in hepatocytes in an FA dose-dependent manner via UCP2.

**Figure 8:**
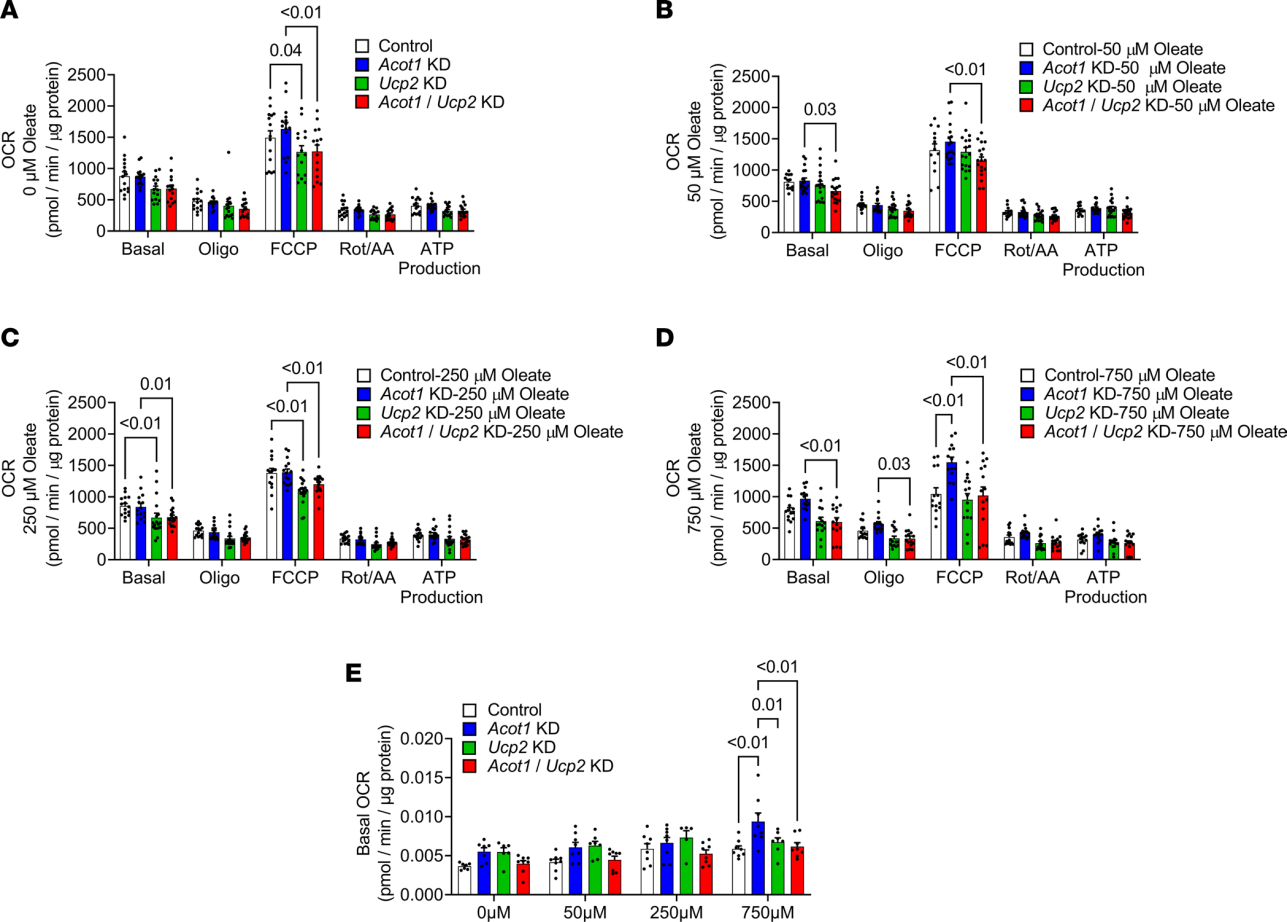
ACOT1 KD–induced increases in oxygen consumption during lipid loading are mediated via increased UCP2. (**A**–**D**) Seahorse mitochondrial stress test time course and the average OCR in AML12 cells either under normal growth media conditions (**A**, *n* = 14–15 per group) or after 24 hours of oleate lipid loading at 50 μM oleate (**B**, *n* = 13–18 per group), 250 μM oleate (**C**, *n* = 15–18 per group), and 750 μM oleate (**D**, *n* = 14–15 per group). (**E**) Basal OCR in AML12 cells was measured over an approximately 22- to 24-hour time period with a Resipher system (*n* = 5–8 per group). All data are presented as mean ± SEM. ANOVA was used for the statistical tests.

ACOT1KO mice fed the HFD had modestly increased oxygen consumption, and this effect was associated with increased hepatic UCP2 abundance. However, the role of UCP2 in modulating oxygen consumption and energy expenditure is controversial. Therefore, it was next tested if KD of UCP2 in the setting of ACOT1 deficiency and FA surplus would attenuate increases in oxygen consumption in hepatocytes. A lentivirus was used and was effective at reducing *Acot1*, *Ucp2*, or both *Acot1* and *Ucp2* mRNA levels in AML12 hepatocytes (Supplemental Figure 6E). A Seahorse mitochondrial stress test revealed that maximal FCCP-stimulated respiration rates were reduced to a similar degree with *Ucp2* KD alone or in combination with *Acot1* KD (Figure 8A). Next, a dose-response experiment was performed to examine how a 24-hour preincubation of different doses of FAs impacted subsequent OCR in the setting of *Acot1* and/or *Ucp2* KD. A 50 μM oleate preincubation reduced both basal and maximal FCCP-stimulated respiration rates in *Acot1*/*Ucp2*-KD cells compared with *Acot1*-KD cells only (Figure 8B), while basal and maximal FCCP-stimulated respiration rates in *Ucp2*-KD and *Acot1*/*Ucp2*-KD cells were equally reduced with a 250 μM preincubation (Figure 8C). However, the addition of 750 μM oleate tended to increase basal respiration rates (*P* = 0.06) in *Acot1*-KD cells compared with control cells and resulted in a statistically significant increase in maximal FCCP-stimulated respiration rates in *Acot1*-KD cells compared with control cells, while double KD of *Acot1*/*Ucp2* prevented this FA-induced increase in basal and maximal oxygen consumption (Figure 8D), suggesting that only very high doses of exogenous FAs in the setting of *Acot1* KD increases oxygen consumption via UCP2. The Seahorse mitochondrial stress test only measured OCR over a short period and in Seahorse media without added FAs. Because of these limitations, a Resipher system was next used to measure OCR over approximately 22 to 24 hours with different doses of FAs in the media (Figure 8E). This experiment revealed that only 750 μM oleate significantly increased basal OCR in *Acot1*-KD AML12 cells, while double KD of *Acot1* and *Ucp2* negated this effect (Figure 8E). However, there was no impact of *Acot1* and/or *Ucp2* KD on 4-HNE protein abundance or dihydroethidium (DHE) fluorescence (superoxide indicator) in the same cells (Supplemental Figure 7) suggesting *Acot1* KD has less of an impact on oxidative stress in vitro in cells. Together, these data indicate that hepatic *Acot1* KD in the setting of high FA levels may increase energy expenditure via UCP2, and, thus, modestly attenuate HFD-induced weight gain.

## Discussion

Dysregulated lipid metabolism has a significant role in many metabolic diseases, such as cardiovascular disease, diabetes, and obesity. Free FAs in all tissues must be activated by long-chain acyl-CoA synthetases into fatty acyl-CoAs before their subsequent metabolism (3), a reaction that is reversible via the action of ACOT enzymes. HFD and obesity (8) or type 2 diabetes (11) are associated with upregulated ACOT1 abundance, suggesting ACOT1 may have a role in the development of obesity and, thus, may be a potential therapeutic target. However, the impact of global ACOT1KO on energy metabolism in various tissues is not well understood. The main finding of this study is that global ACOT1KO modestly attenuates HFD-induced weight gain by increasing energy expenditure.

The role of UCP2 in energy expenditure is controversial and still debated (16, 17). Studies in hepatocytes (18) or adipocytes (14) show that overexpression of UCP2 reduces mitochondrial membrane potential and ATP generation, effects that are consistent with an increase in mitochondrial uncoupling, although total energy expenditure was not measured. Variants of UCP2 that cause reduced protein expression and/or activity in humans is associated with obesity depending upon ethnicity and sex (19) and lower energy expenditure (20, 21). In humans, s.c. UCP2 expression is associated with higher weight loss in those on a hypocaloric diet (22). Consistent with these effects on energy balance, both male and female UCP2 KO mice gain more weight when fed an HFD (23–29). A seminal finding in the field was that UCP2 binds FAs and “flips” them across the inner mitochondrial membrane to facilitate proton leak (30). In this FA-cycling model, UCP2 binds unprotonated FAs on the matrix side of the inner mitochondrial membrane and facilitates the flip of the FA to the lateral side of the membrane (facing the inner membrane space). Upon release from UCP2, the FA then diffuses laterally and becomes protonated (H^+^ addition), which allows the FA to spontaneously flip back to the matrix side of the membrane where the H^+^ is subsequently released (i.e., proton leak). The unprotonated FA can then bind UCP2 again and restart the cycle. This model is supported by the fact that UCP2 is activated by and requires FAs for its proton transport functions (30, 31). To further test if UCP2 was contributing to the increase in energy expenditure observed with ACOT1KO and HFD in our model, ACOT1 and/or UCP2 were genetically silenced in adipocytes or hepatocytes under different FA loads. These experiments revealed that ACOT1 deficiency during lipid loading in hepatocytes, but not adipocytes, increases energy expenditure via UCP2. These findings suggest that the ability of UCP2 to increase energy expenditure may be tissue dependent as well as FA load dependent. Moreover, these data provide a plausible mechanism by which ACOT1KO increases energy expenditure to attenuate weight gain during HFD feeding through the interplay between ACOT1 and UCP2 to regulate oxygen consumption under the setting of a high lipid load. The putative mechanism by which ACOT1 deficiency activates UCP2 in our model is unknown. It could be due to an increase in FAs. Additionally, given that CoAs bind UCP2 (32), it could be that ACOT1 deficiency leads to the accumulation of fatty-acyl-CoAs, which may in turn bind to and activate UCP2, although we have no direct evidence to support this potential mechanism.

The liver utilizes activated FAs for energy metabolism, and when FA flux increases, such as during fasting, ACOT1 is induced and acts to deactivate FAs, which are destined for oxidative pathways, thereby reducing acyl-CoA levels, which are potentially toxic metabolites (7–9). In line with our previous work (7), loss of ACOT1 increased FA oxidation as assessed indirectly by an increase in circulating levels of the ketone body beta-hydroxybutyrate as well as an increase in hepatic acetyl-CoA levels. Additionally, ACOT1KO mice fed the HFD had reduced electron transport chain complex I and V protein abundance, suggesting a reduction in select aspects of mitochondrial content, although mtDNA content was not altered. These findings corroborate our previous data with acute hepatic ACOT1KO in the liver, where increased FA flux was associated with a reduction in some markers of mitochondrial content (7). Together, these data suggest that when hepatic FA availability increases with the HFD, more FAs are activated than can be oxidized, and in this scenario, ACOT1 may act as a brake to slow FA oxidation and prevent mitochondrial damage and, presumably, loss of select components of mitochondrial content due to excessive FA activation.

One of the detriments caused by excessive mitochondrial FA oxidation includes the generation of ROS that damages cellular lipids, DNA, RNA, and proteins. To prevent excessive damage from ROS, numerous antioxidant and uncoupling enzymes are expressed in various cellular locations. The major antioxidant enzyme in the liver is GSH, and despite a reduction in oxidative stress in the liver of ACOT1KO mice, this was not associated with alterations in GSH levels. Instead, UCP2, which can reduce oxidative stress by uncoupling the proton gradient (33, 34), was upregulated with ACOT1KO in both WAT and the liver but only in the setting of an HFD, which is consistent with our prior work, which showed that acute hepatic ACOT1 KD increased hepatic UCP2 (7). Contrary to our prior work, herein we find long-term ACOT1KO in the liver reduced oxidative stress during HFD feeding, while our previous report showed that short-term ACOT1KO in the liver promoted oxidative stress during the LFD (7). In the present investigation, we found that short-term ACOT1 KD did not alter oxidative stress in cells, suggesting that the impact of ACOT1 deficiency may depend on the duration and extent of the KO in addition to the model system utilized.

In conclusion, the data presented in the current study suggest that ACOT1 inhibition attenuates BW gain during diet-induced obesity by increasing energy expenditure through UCP2 in hepatocytes. Despite attenuated fat gain with ACOT1 inhibition, there was no effect on whole-body glucose tolerance or insulin sensitivity suggesting that ACOT1 may uncouple changes in weight and adiposity with insulin and glucose homeostasis. Nevertheless, pharmacological agents that inhibit ACOT1 may have clinical utility in preventing or treating obesity or diseases with alterations in lipid metabolism.

## Methods

### Animals.

All mice were housed in a controlled temperature (20°C–22°C) and light (14-hour light/10-hour dark) vivarium during these studies. ACOT1 heterozygous KO mice were purchased from the NIH Knockout Mouse Project (VG12838), which uses VelociGene’s KOMP Allele Genotyping Strategies (Figure 1A). Next, heterozygous mice were bred together to generate mice with homozygous whole-body ACOT1KO. To confirm ACOT1 KO) via PCR, DNA was isolated from tail clips using DNeasy blood and tissue kit from QIAGEN and genotyped based on PCR protocols provided by the NIH Knockout Mouse Project using primers to identify the gene (TDF: AAGGATGAGACTATACCCCCTGTGA and SDR: ACTTCGGGAGGGAAGATGAG) and for the cassette (SUF: GTCAAAGACTGCCGGCTAAG and LacZRev: GTCTGTCCTAGCTTCCTCACTG), which indicates the absence of the ACOT1 gene (Figure 1B). Mice had free access to water and food, and at 8 weeks of age, ACOT1KO or littermate WT mice were switched to either a purified LFD (TD.94045) or a 45% HFD (TD.06415) from Harlan Teklad for 12 weeks. ACOT1 is highly active in most tissues during fasting (8); thus, mice in this study were fasted overnight for approximately 16 hours before sacrifice.

### Cell culture.

AML12 cells (ATCC, catalog CRL-2254) were grown in complete DMEM/F12 (1:1) media (Thermo Fisher Scientific, catalog 11320082) while HEK293T (ATCC, catalog CRL-3216) cells were grown in growth media (10% FBS, 1% Pen-Strep, and 4.5 g/L glucose with l-glutamine DMEM; Thermo Fisher Scientific, catalog 11965-092). The 293T cells, along with the packaging plasmids psPAX2 (Addgene, catalog 12260) and envelope expressing plasmid pMD2.G (Addgene, catalog 12259) were used to generate either the shACOT1 (Sigma, catalog TRCN0000033253) or shUCP2 (Sigma, catalog TRCN0000349467) lentivirus, as previously described by our lab (35). The 3T3-L1 or stromal vascular cells were initially supplied with media containing 10% bovine calf serum (BCS) (Thermo Fisher Scientific, catalog 16010159), 1% Pen-Strep, and 4.5 g/L glucose with l-glutamine and sodium pyruvate DMEM (Thermo Fisher Scientific, catalog 11995065). To differentiate the 3T3-L1 or stromal vascular cells into adipocytes, initially the cells were incubated with differentiation media 1 (10% FBS, 1% Pen-Strep, 4.5 g/L glucose with l-glutamine, sodium pyruvate with 1 μM rosiglitazone, 1 μM DEXA, 500 μM IBMX, and 100 nM insulin) for days 0–2 of differentiation. For days 3–6 of differentiation, the cells were incubated in differentiation media 2 (10% FBS, 1% Pen-Strep, 4.5 g/L glucose with l-glutamine, sodium pyruvate with 1 μM rosiglitazone, and 500 nM insulin). For days 7 and up of differentiation, the cells were incubated with differentiation media 3 (10% FBS, 1% Pen-Strep, 4.5 g/L glucose with l-glutamine, and sodium pyruvate with 500 nM insulin) until cells were differentiated into adipocytes, and then experiments were performed.

### Stromal vascular cell isolation.

Mice were euthanized and sterilized in 75% alcohol for 5 minutes. Both gWAT and iWAT were isolated and then minced into 3–4 mm pieces in Krebs Ringer Bicarbonate HEPES (KRBH) buffer (1.2 M NaCl, 40 mM KH_2_PO_4_, 10 mM MgSO_4_.7H_2_O, 10 mM CaCl_2_, 100 mM NaHCO_3_, 300 mM HEPES, and 0.1 mg/mL gentamicin sulfate). Next, minced tissue was placed in a 50 mL Falcon tube (Corning), fresh collagenase was added (1.7 mg/mL) to the KRBH buffer, and the tube was incubated in a water bath at 37°C for 1.5 hours. After digestion, the sample was poured through a 200 μm mesh filter and then centrifuged at 114*g* for 1 minute. The buffer under the floating cells was collected, centrifuged again at 114*g* for 1 minute, and the buffer under the floating cells was collected and poured through a 22 μm filter. The remaining buffer was centrifuged at 257*g* for 5 minutes to pellet the stromal vascular cells. The cells were washed once with 10% BCS medium, plated on cell culture plates, and differentiated into adipocytes before experiments.

### Resipher.

Basal respiration rates over 22–24 hours were measured using a Resipher system (Lucid Scientific) according to manufacturer specifications in a 5% CO_2_ incubator. Approximately 5,000 AML12, stromal vascular, or 3T3-L1 cells were initially plated in a tissue culture-treated Nunc 96-well plate (Thermo Fisher Scientific, catalog 167008). For AML12 cells, experiments started the next day, whereas stromal vascular cells and 3T3-L1 cells were differentiated before measuring OCR. The media (100 μL per well) used for AML12 cells was 10% FBS with various amounts of oleate conjugated to BSA added. The media (100 μL per well) used for stromal vascular or 3T3-L1 cells was differentiation media 3 with various amounts of oleate conjugated to BSA added.

### Seahorse.

Basal and maximal OCR were measured using a Seahorse XFe96 Analyzer along with the Mitochondrial Stress Test Kit (Agilent, catalog 103015-100), as described previously by our lab (35).

### Indirect calorimetry.

The Oxymax Comprehensive Lab Animal Monitoring System by Columbus Instruments was used to measure oxygen consumption, carbon dioxide production, RER, physical activity levels, and food intake in mice with free access to food or during a 24-hour fast. This experiment occurred in a subset of mice at 18 weeks of age (week 10 of diet). The RER was generated from formulas provided by the manufacturer.

### Hepatic and serum triglyceride analysis.

Hepatic triglycerides were extracted using the chloroform/methanol extraction method. Extracted or serum triglycerides were measured using a colorimetric assay kit (Stanbio Laboratory) as described previously by our lab (36).

### Serum free FA and ketone bodies.

Serum non-esterified FAs were determined by the Wako HR Series NEFA-HR (2) kit. Serum total ketone bodies (acetoacetone + 3 hydroxybutyrate) were assessed using a cyclic enzymatic assay (FUJIFILM Wako Diagnostic).

### Lipidomics.

The Emory Integrated Lipidomics Core at Emory University extracted and measured acyl-CoA levels. Briefly, the tissue was weighed, placed in PBS, and the tissue was ruptured using a Bead Ruptor 24 Elite bead mill homogenizer (Omni International) with 1.4 mm ceramic beads (Thermo Fisher Scientific). Homogenates were centrifuged at 15,000 RPM for 5 minutes at 4°C and the acyl-CoA–enriched supernatants were collected and measured using LC-MS/MS on an Agilent Infinity 1250 II/6495c Triple Quadrupole Mass Spectrometer. Mass Hunter 10.1 analysis software was used to determine acyl-CoA levels.

### RNA isolation and analysis.

RNA was isolated using the TRIzol Reagent (Thermo Fisher Scientific) and cDNA was made using SuperScript VILO (Invitrogen). Real-time quantitative PCR (qPCR) was performed with SYBR Select reagents (Thermo Fisher Scientific) on an Applied Biosystems StepOnePlus qPCR system. The reverse ACOT primer used was 5′ ATGATCTGGGGCTTCTCCTT 3′ while the forward ACOT primer used was 5′ GATGGCCTCAAGGATGTTGT 3′. RPL32 was used as a housekeeping gene. mtDNA content was measured using the following primers: 16s rRNA forward primer: CCGCAAGGGAAAGATGAAAGAC; 16s rRNA reverse primer: TCGTTTGGTTTCGGGGTTTC; ND1 forward primer: CTAGCAGAAACAAACCGGGC; ND1 reverse primer: CCGGCTGCGTATTCTACGTT; HK2 forward primer: GCCAGCCTCTCCTGATTTTAGTGT; and HK2 reverse primer: GGGAACACAAAAGACCTCTTCTGG. The mtDNA content was expressed as a ratio between mitochondrially encoded DNA (either 16S rRNA or ND1) over nuclear encoded DNA (HK2).

### Western blotting.

Protein was isolated from tissues via homogenization in lysis buffer and the protein concentration was quantified using the Pierce BCA Protein Assay Kit (Thermo Fisher Scientific). ACOT1 abundance and knockout were determined by Western blot with a custom ACOT1/ACOT2 Ab, which was developed in rabbit by injecting the antigen CSVAAVGNTISYKDET-amide (21^st^ Century Biochemicals). The primary Abs SIRT1 (3931S), UCP2 (89326S), and ATGL (2138S) were purchased from Cell Signaling Technology. The rodent OxPhos (ab110413), UCP1 (ab10983), and 4-HNE (ab48506) primary Abs were purchased from Abcam. The appropriate LI-COR secondary Abs (925-32212 and 925-32211) were used while fluorescence blots were obtained using a LI-COR Odyssey Fc imaging system.

### Oral glucose tolerance test.

Glucose tolerance was assessed at 10 weeks on diet, after an overnight fast (~16 hours). Tail vein blood glucose was taken at baseline and at 15, 30, 60, and 90 minutes after an oral gavage of a dextrose solution (2 g/kg BW).

### Serum insulin.

Serum insulin was measured using a commercially available ELISA kit (Crystal Chem) as previously described (37). Insulin resistance was calculated using the HOMA-IR equation (basal glucose [mg/dL] × basal insulin [mU/mL]/405) (38).

### Tissue imaging.

The preparation and imaging of tissue sections were performed as previously described by our lab (39–41). Briefly, tissue sections were fixed in 10% formalin and subsequently paraffin-embedded for H&E staining, which was performed by the University of Minnesota’s BioNet Histology Core. To measure superoxide production (oxidative stress) in AML12 cells, fluorescence imaging was performed using the superoxide indicator DHE (Abcam, catalog ab236206) and an ECHO fluorescence microscope and fluorescence plate reader. Briefly, live cells were incubated with 15 μM of DHE for 1.5 hours. NucBlue Live ReadyProbes (Thermo Fisher Scientific, catalog R37605) was added during the final 20 minutes to stain for nuclei. After staining with DHE and NucBlue, the cells were washed once, and stain-free media was added before imaging or reading on the plate reader.

### Thioesterase activity assay.

Thioesterase activity was determined in male mice fed a purified diet. Frozen liver samples were homogenized and centrifuged at 100,000*g* for 1 hour at 4°C, after which supernatants were collected and 1 mg protein in assay buffer (50 mM KCl, 10 mM HEPES) was added to each well. DTNB was added at a working concentration of 50 μM and read at 405 nm at 37°C. Palmitoyl CoA was added at 20 μM and read at 405 nm at 37°C for 3–5 minutes.

### Statistics.

Statistical analyses were performed with GraphPad Prism 8.1.1 software, IBM SPSS statistical software, or CalR software (42). Values are expressed as the mean ± SD unless otherwise noted. Two-way ANOVA with appropriate post hoc testing was used to test for statistical significance. A *P* value less than or equal to 0.05 was considered significant. For the indirect calorimetry analysis, ANCOVA with body mass as the covariate was performed using CalR (42) and SPSS software.

### Study approval.

All protocols used during this study were approved by the IACUC of the University of Minnesota.

### Data availability.

Values for all data points shown in the figures can be found in the supplemental material Excel file titled “Supporting Data Values.” Data for each separate figure panel are listed in individual tabs within the Excel file.

## Author contributions

TDH performed conceptualization, methodology, validation, formal analysis, investigation, data curation, writing of the original draft, visualization, and project administration. MPF performed validation, investigation, and reviewing and editing. CD performed validation, investigation, and reviewing and editing. MTM performed validation, investigation, reviewing and editing, and project administration. CC performed investigation and reviewing and editing. DGM performed conceptualization, methodology, resource provision, reviewing and editing, supervision, project administration, and funding acquisition.

## Supplementary Material

Supplemental data

Supporting data values

## Version 1 08/10/2023

In-Press Preview

## Version 2 09/22/2023

Electronic publication
