# Supplementary material for: ACOT1 deficiency attenuates high-fat diet–induced fat mass gain by increasing energy expenditure
Source: JCI Insight. 2023 Sep 22;8(18):e160987. doi: 10.1172/jci.insight.160987 (PMC10561717; doi:10.1172/jci.insight.160987)
Supplement: Supplemental data [file jciinsight-8-160987-s171.pdf]

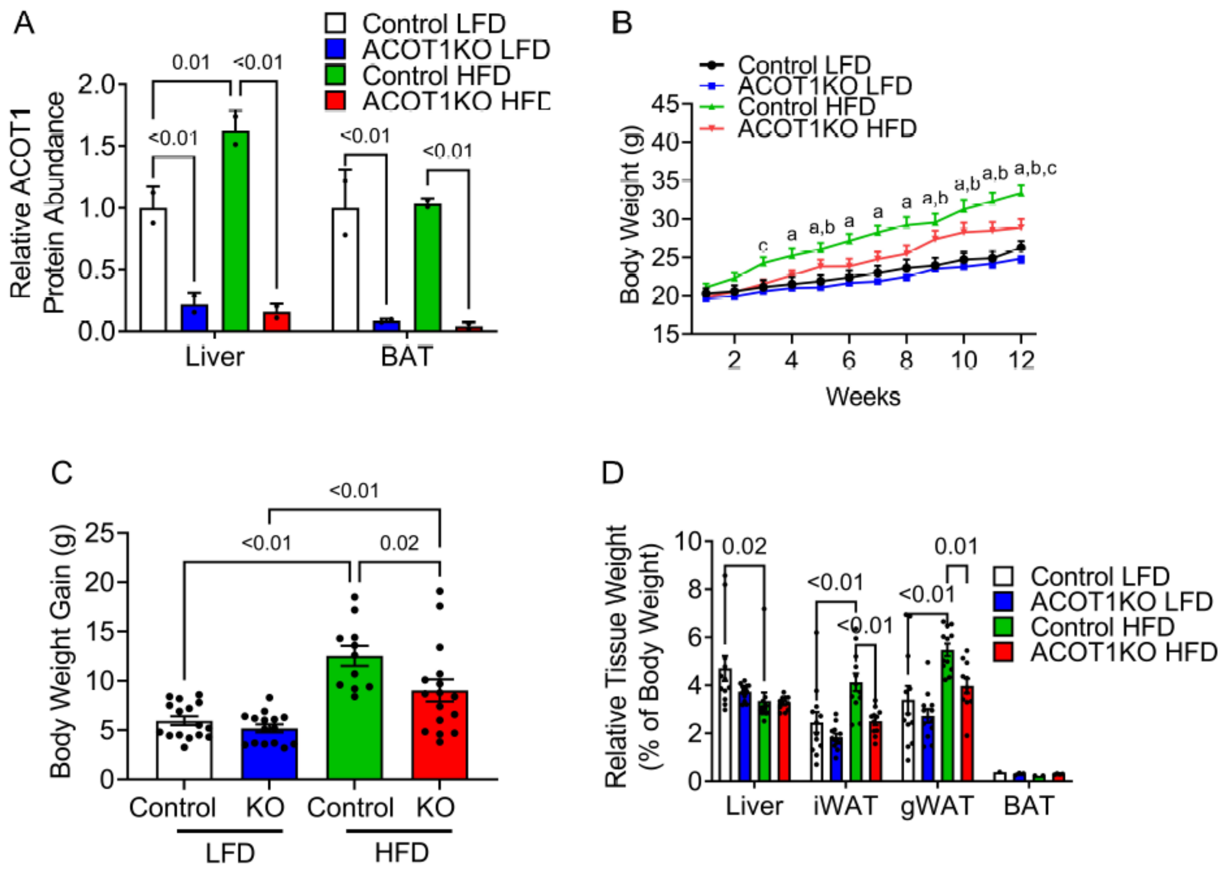

Supplemental Figure 1. Protein densitometry and female ACOT1KO mice. (A) Protein densitometry quantification from Figure 1D (n = 2 per group). (B) Body weight changes in female mice (n = 12-16 per group). (C) Body weight gain (calculated as the difference between final body weight and initial body weight in 20-week-old female mice given the LFD or HFD for 12 weeks (n = 11-16 per group). (D) Relative tissue weight of various female tissues (n = 1-12 per group). All data are presented as means ± S.E.M. $^{a}P \leq 0.01$ control LFD vs. control HFD, $^{b}P = 0.02$ ACOT1KO LFD vs. ACOT1KO HFD, $^{c}P \leq 0.01$ control HFD vs. ACOT1KO HFD. ANOVAs were used for statistical tests.

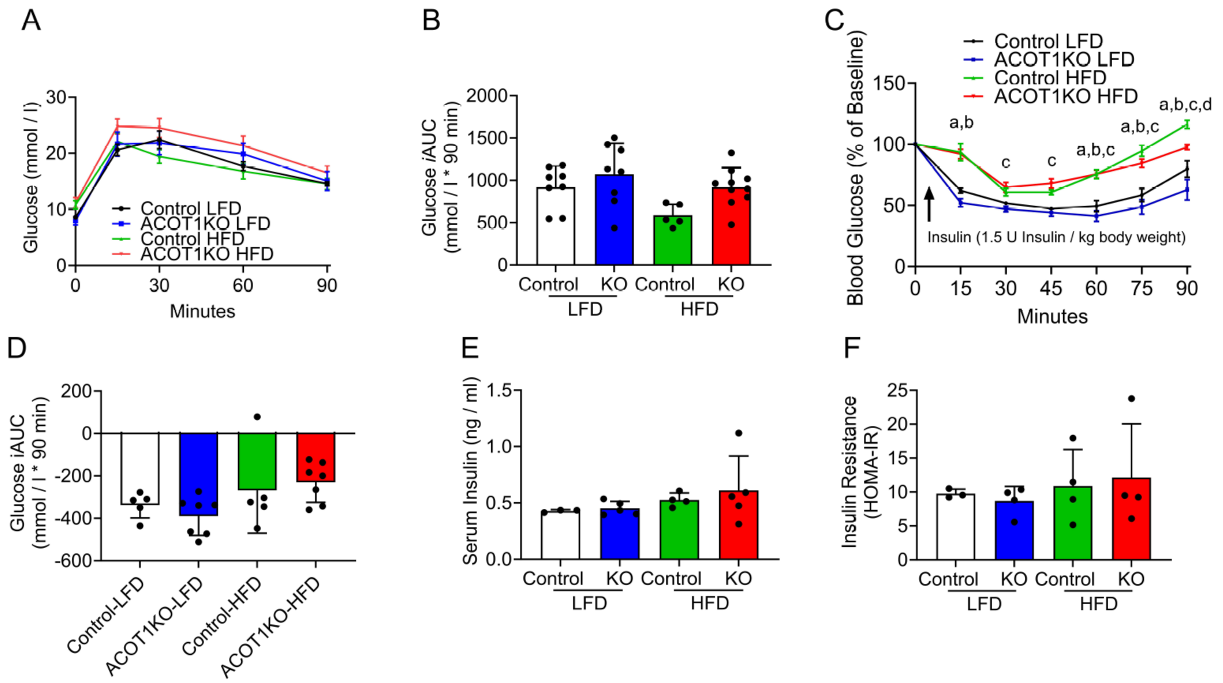

Supplemental Figure 2. No impact of ACOT1 deficiency on glucose metabolism or insulin resistance in male mice. (A) Glucose time course during an oral glucose tolerance test in 20-week-old male mice given the LFD or HFD for 12 weeks. (n = 5-10 per group). (B) Glucose incremental area under the curve (n = 5-10 per group). (C) Glucose time course during an insulin tolerance test (n = 4-7 per group). (D) Glucose incremental area under the curve during insulin tolerance test (n = 5-7). (E) Fasting serum insulin levels (n = 3-5 per group). (F) Insulin resistance was calculated using the Homeostatic Model for Insulin Resistance (HOMA-IR) formula (n = 3-4 per group). All data are presented as means ± S.E.M. $^{a}P \leq 0.01$ control LFD vs. control HFD, $^{b}P = 0.02$ ACOT1KO LFD vs. ACOT1KO HFD, $^{c}P \leq 0.01$ ACOT1KO LFD vs. ACOT1KO HFD, $^{d}P = 0.02$ control HFD vs. ACOT1KO HFD. ANOVAs were used for statistical tests.

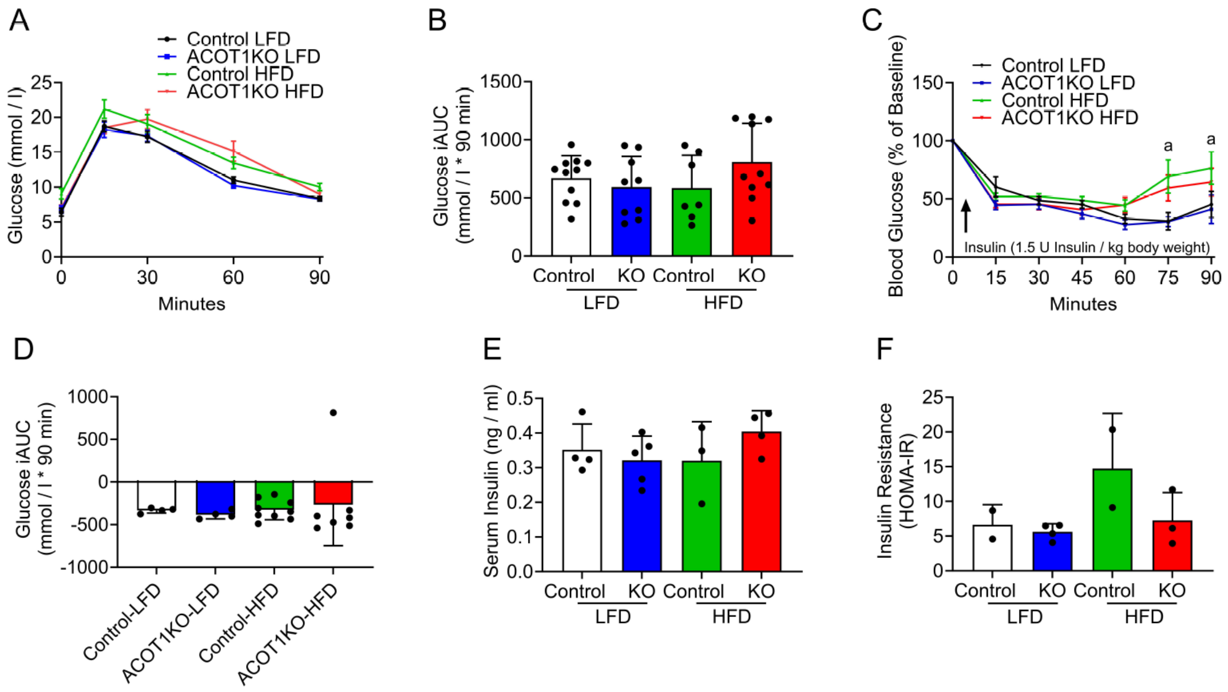

Supplemental Figure 3. Glucose tolerance and insulin sensitivity in female mice. (A) Glucose time course during an oral glucose tolerance test in 20-week-old female mice given the LFD or HFD for 12 weeks. (n = 7-11 per group). (B) Glucose incremental area under the curve (n = 7-11 per group). (C) Glucose time course during an insulin tolerance test (n = 4-9 per group). (D) Glucose incremental area under the curve during insulin tolerance test (n = 4-9). (E) Fasting serum insulin levels (n = 3-5 per group). (F) Insulin resistance was calculated using the Homeostatic Model for Insulin Resistance (HOMA-IR) formula (n = 2-4 per group). All data are presented as means ± S.E.M. [a]P = 0.04 control LFD vs control HFD. ANOVAs were used for statistical tests.

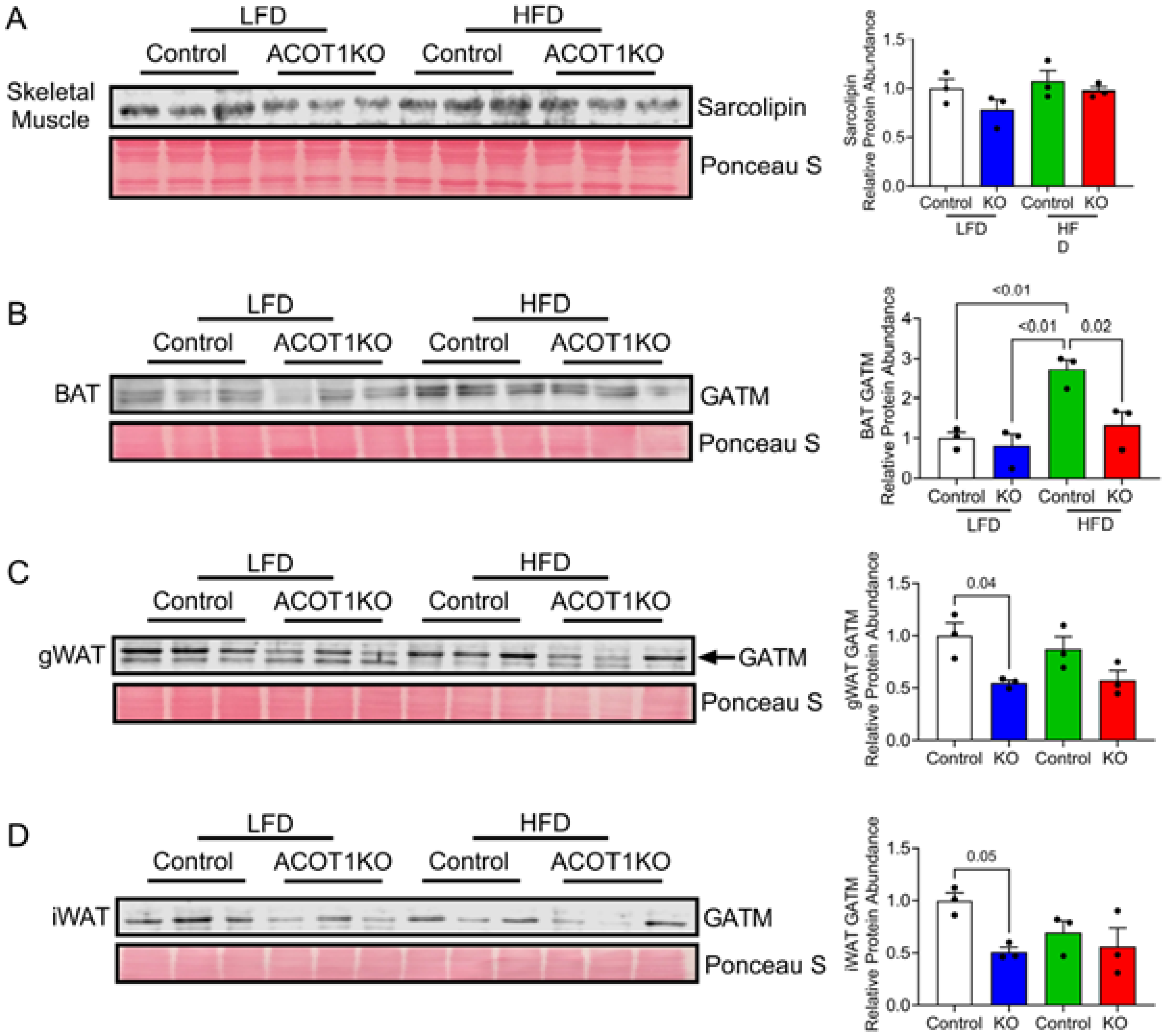

Supplemental Figure 4. Abundance of proteins involved in UCP1 independent mechanisms of thermogenesis. (A) Western blot image (left) and densitometry quantification (right) of sarcolipin protein abundance in skeletal muscle from 20-week-old control or ACOT1KO mice fed the LFD or HFD for 12 weeks (n = 3 per group). (B-D) Western blot image (left) and densitometry quantification (right) of arginine:glycine amidinotransferase (GATM) protein abundance in BAT (B), gWAT (C), and iWAT (D) (all n = 3 per group). All data are presented as means ± S.E.M. ANOVAs were used for statistical tests.

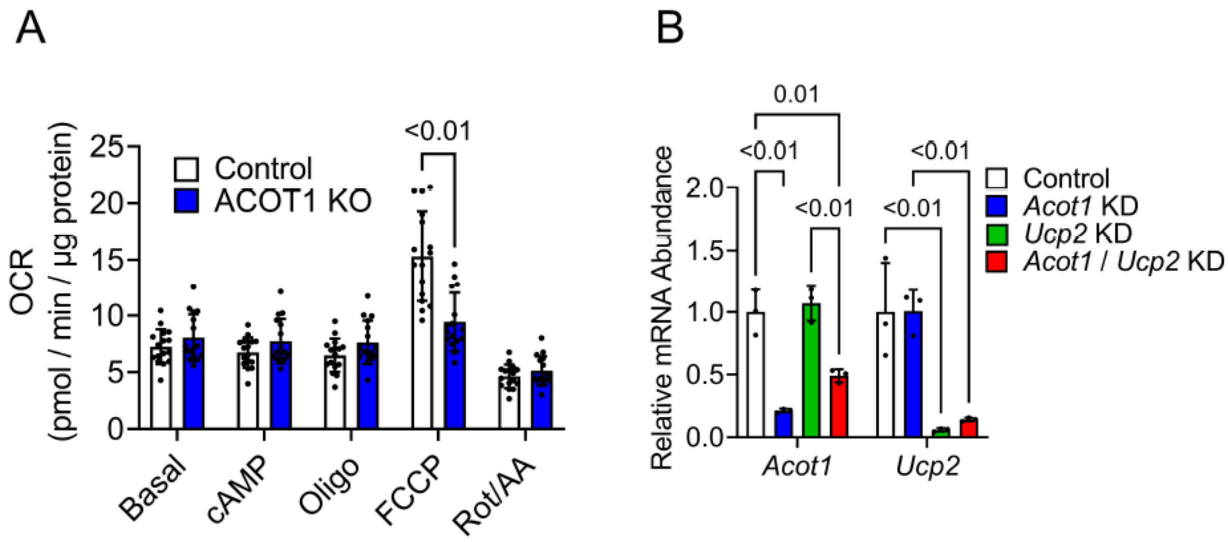

Supplemental Figure 5. Oxygen consumption rates and mRNA abundance in adipocytes. (A) OCR in stromal vascular cells was measured with a Seahorse under basal conditions and after sequential injections of 8-bromo cyclic adenosine monophosphate (cAMP), oligomycin (oligo), carbonyl cyanide-p-trifluoromethoxyphenyl hydrazone (FCCP), and rotenone/antimycin A (Rot/AA) (n = 16 per group). (B) Relative mRNA abundance of *Acot1* or *Ucp2* in 3T3-L1 cells treated with control (scrambled), sh*Acot1*, sh*Ucp2*, or both sh*Acot1* / sh*Ucp2* lentivirus to induce gene knockdown (KD) (n = 3 per group). All data are presented as means ± S.E.M. ANOVAs were used for statistical tests.

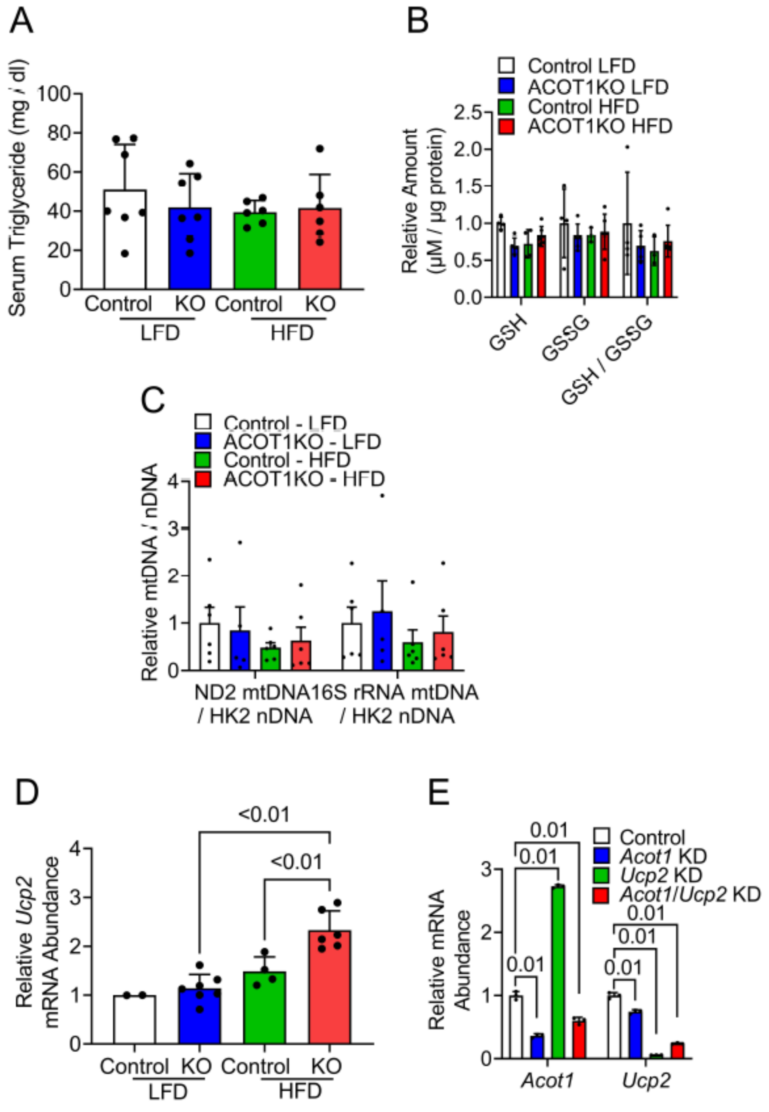

Supplemental Figure 6. ACOT1 deficiency and hepatic metabolism. (A) Serum triglyceride levels in 20-week-old mice fed the LFD or HFD for 12 weeks (n = 6-7 per group). (B) Relative hepatic glutathione (GSH), oxidized glutathione (GSSG), and the GSH / GSSG ratio (n = 3-6 per group). (C) Relative hepatic mtDNA content (n = 5-6 per group). (D) Relative mRNA abundance of hepatic *Ucp2* (n = 2-7 per group). (E) Relative mRNA abundance of *Acot1* or *Ucp2* in AML12 cells treated with control (scrambled), sh*Acot1*, sh*Ucp2*, or both sh*Acot1* / sh*Ucp2* lentivirus to induce gene knockdown (KD) (n = 3 per group). All data are presented as means ± S.E.M. ANOVAs were used for statistical tests.

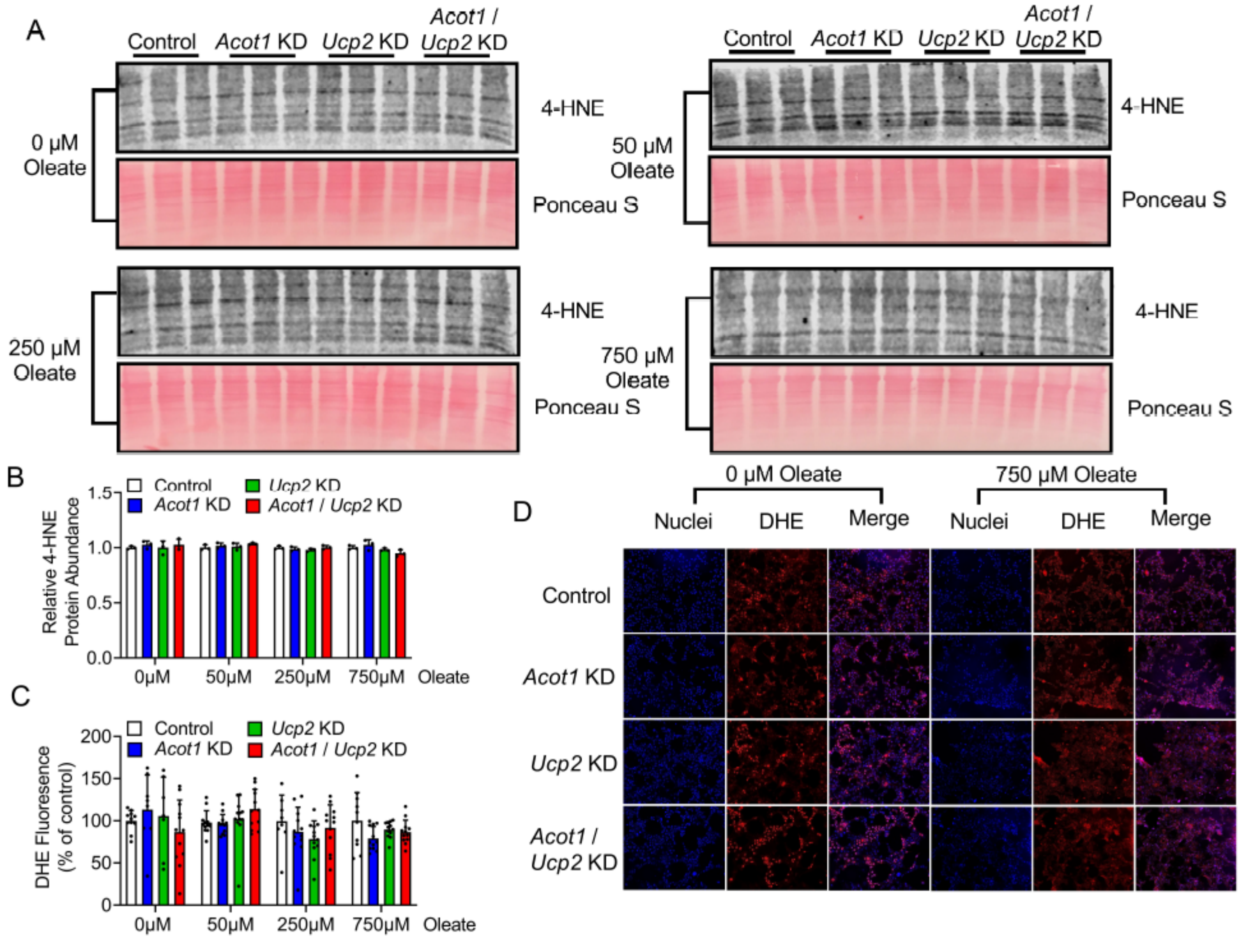

Supplemental Figure 7. Oxidative stress in AML12 cells treated with various doses of oleate for 24 hours. (A-B) Western blot images (A) and densitometry quantification (B) of 4-HNE protein abundance in AML12 cells treated with control (scrambled), sh*Acot1*, sh*Ucp2*, or both sh*Acot1* / sh*Ucp2* lentivirus to induce gene knockdown (KD) and after a 24 h incubation with the indicated amount of oleate (n = 3 per group, per condition). (C-D) Dihydroethidium (DHE) fluorescence (n = 7-12 per group) (C) and representative images (D) of select conditions of AML12 cells treated with the indicated lentivirus and pre-treated with oleate (10X objective). A total of 3-4 images per condition were taken. All data are presented as means ± S.E.M. ANOVAs were used for statistical tests.
